# Irrational antibiotic use among teachers and academic staff, Shiraz, Iran

**DOI:** 10.1186/1753-6561-5-S6-P153

**Published:** 2011-06-29

**Authors:** M Askarian, N Maharlouie

**Affiliations:** 1 Community Medicine, Shiraz University of Medical Sciences, Health Policy Research Center, Shiraz, Iran, Islamic Republic Of; 2 Shiraz University of Medical Sciences, Health Policy Research Center, Shiraz, Iran, Islamic Republic Of

## Introduction / objectives

Antibiotics are amongst the most sold drugs. Inappropriate use of antibiotics leads not only to emergence of resistant bacteria, but also economic loss and adverse reactions. Knowledge, attitude and practice of academic staff and high school teachers about the use of antibiotics and self-medication are assessed in this survey. Also, the relationship between these factors is determined.

## Methods

In this cross-sectional survey, 320 academic staff (except physicians, pharmacists and dentists) and 150 high school teachers were questioned by a questionnaire composed of 15 questions to assess their knowledge, attitude and practice about the use of antibiotics and self-medication. The reliability of the questionnaire was assessed by Crohnbach’s alpha internal consistency coefficient and the results were analyzed, using Mann-Witnney U. Spearman’s Correlation Coefficient was used to determine the correlation between knowledge, attitude and practice.

## Results

The questionnaires were completed by 134 academic staff and 308 high school teachers, among whom, respectively, 35.8% and 47.1% had had self-medication during the previous year, mostly to relieve sore throat in both groups. The teachers were significantly better than the faculty staff in knowledge (P= 0.008) and practice (P<0.001).

In both teachers group and academic staff, a direct linear poor relationship was detected between attitude and practice (r = 0.243 & r = 0.238, P < 0.01), and a poor reverse linear relationship was seen between knowledge and practice (P= -.0218) in the teachers group.

## Conclusion

According to our results, Self-medication and irrational use of antibiotics is common among highly educated people in the community in Iran, which can be prevented by improving their knowledge.

## Disclosure of interest

None declared.

